# Features of Microglia and Neuroinflammation Relevant to Environmental Exposure and Neurotoxicity

**DOI:** 10.3390/ijerph8072980

**Published:** 2011-07-20

**Authors:** Andrew D. Kraft, G. Jean Harry

**Affiliations:** 1 Oak Ridge Institute for Science and Education Research Participant at the U.S. Environmental Protection Agency (EPA)/National Center for Environmental Assessment, Office of Research and Development, U.S. EPA, Arlington, VA 22202, USA; E-Mail: kraft.andrew@epamail.epa.gov; 2 Neurotoxicology Group, Laboratory of Toxicology and Pharmacology/National Institute of Environmental Health Sciences, National Institutes of Health, Research Triangle Park, NC 27709, USA

**Keywords:** neuroinflammation, microglia, neurotoxicity, neurodegeneration, cytokines, environmental exposure

## Abstract

Microglia are resident cells of the brain involved in regulatory processes critical for development, maintenance of the neural environment, injury and repair. They belong to the monocytic-macrophage lineage and serve as brain immune cells to orchestrate innate immune responses; however, they are distinct from other tissue macrophages due to their relatively quiescent phenotype and tight regulation by the CNS microenvironment. Microglia actively survey the surrounding parenchyma and respond rapidly to changes such that any disruption to neural architecture or function can contribute to the loss in regulation of the microglia phenotype. In many models of neurodegeneration and neurotoxicity, early events of synaptic degeneration and neuronal loss are accompanied by an inflammatory response including activation of microglia, perivascular monocytes, and recruitment of leukocytes. In culture, microglia have been shown to be capable of releasing several potentially cytotoxic substances, such as reactive oxygen intermediates, nitric oxide, proteases, arachidonic acid derivatives, excitatory amino acids, and cytokines; however, they also produce various neurotrophic factors and quench damage from free radicals and excitotoxins. As the primary source for pro-inflammatory cytokines, microglia are implicated as pivotal mediators of neuroinflammation and can induce or modulate a broad spectrum of cellular responses. Neuroinflammation should be considered as a balanced network of processes whereby subtle modifications can shift the cells toward disparate outcomes. For any evaluation of neuroinflammation and microglial responses, within the framework of neurotoxicity or degeneration, one key question in determining the consequence of neuroinflammation is whether the response is an initiating event or the consequence of tissue damage. As examples of environmental exposure-related neuroinflammation in the literature, we provide an evaluation of data on manganese and diesel exhaust particles.

## 1. Introduction

Neuroinflammation represents the coordinated cellular response to tissue damage. While the appropriate regulation of this process facilitates recovery, uncontrolled neuroinflammation can induce secondary injury. Microglia serve as the resident mononuclear phagocytes of the brain and are highly heterogeneous within the healthy CNS. They comprise only 10% of the total cell population of the brain. However, they exhibit multiple morphological phenotypes and, presumably, multiple functional profiles depending on their environment [1,2]. Structurally, microglia display a dynamic and active phenotype with ongoing retraction and extension of processes into the brain parenchyma [3]. This supports the idea of a surveillance function for microglia in the healthy brain and indicates that these cells are poised to rapidly respond to environmental changes. This concept has been supported by the observation that microglial activation is likely an early event in all forms of pathology. In the human brain, microglial activation and neuroinflammation have been associated with viral or bacterial infection, autoimmune disease such as multiple sclerosis, head trauma, vascular system damage, neuropsychiatric disorders, and neurodegenerative diseases. The presence of activated microglia was initially considered as a sensitive marker to identify sites predestined for imminent tissue destruction [4]. Based upon this, a role for microglial activation and neuroinflammation has more recently been considered as an underlying and, possibly, unifying factor of neurotoxicity from environmental exposures.

The normal, adult central nervous system (CNS) parenchyma is an immunoprivileged site [5], given that the resident myeloid cells of the CNS parenchyma, the microglia, are unable to assume the functions of dendritic cells to ingest antigen and stimulate naïve T cells following cell migration to the draining lymph nodes [6]. However, these resident immune cells of the brain maintain a low or undetectable level of immune products at rest and, only upon appropriate stimulation, initiate immune responses. An alternative description of CNS “privilege” is related not to the absolute absence of immunological components, but rather to the complex regulation required for a system with a limited capacity for regeneration, and thus, a requirement to limit cellular damage (for a review, see [4]).

Inflammatory responses are typically localized and involve communication between immune, vascular, and parenchymal cells, with resident tissue macrophages playing key roles as sentinels. In classic inflammatory disorders of the CNS, such as multiple sclerosis (MS), an infiltration of various immune cell subsets from the periphery is evident. These include a broad spectrum of the T and B lymphocytes, dendritic cells, and monocytes that transform into brain macrophages. This cellular reaction includes an innate immune response from non-antigen specific monocytes and neutrophils, as well as an adaptive immune response from antigen-specific T and B lymphocytes. In MS patients and in models of experimental autoimmune encephalomyelitis (EAE), a role for interleukin (IL)-18 and caspase 1 in amplifying Th1 immune responses has been shown [7–9]. In this case, IL-18 can direct autoreactive T cells, induce interferon gamma (IFNγ) release by natural killer cells, and promote autoimmune neurodegeneration [10]. T cells do not persist within the CNS parenchyma unless they are restimulated by antigen previously encountered in the peripheral lymphoid organs [11]. Restimulation of T cells by mononuclear phagocytes of the CNS is the responsibility of non-parenchymal mononuclear phagocytes found in the choroid plexus, meninges, and perivascular spaces [12–17]. This restimulation process and the cytokine signals secreted by T cells are required for non-resident, mononuclear phagocytes to invade the CNS parenchyma [18].

## 2. Receptors for Sensing Potential Threats

Microglia within the CNS parenchyma serve as the resident immune cells and, as such, are sensitive sensors of events occurring within their immediate environment. Thus, they often provide the first line of defense against invading microbes and, *via* interactions with neurons, they frequently are the first to detect critical changes in neuronal activity and health. In addition to biological stimuli, *in vitro* data indicates that exposure to environmental chemicals and compounds with neuropharmacological properties can directly stimulate microglia (e.g., [19–22]). In the healthy brain, microglia are in intimate contact with neurons, for which they serve important developmental support and maintenance functions, such as clearance of aberrant proteins [3,23–27]. Healthy neurons maintain microglia in an inactive state *via* secreted and membrane-bound signals, including CD200, CX3CL1 (fractalkine), neurotransmitters and neurotrophins [28–30]. The expression of CD200 on neurons and endothelial cells in the CNS and the expression of its receptor, CD200R, predominantly on cells of myeloid origin, including macrophages and microglia, support a mechanism of neuronal/glia interactions to maintain microglia in a quiescent state [29,31–34]. In mice deficient for CD200, the microglia exhibit a morphological phenotype of less ramified and shorter processes, an increased expression of CD11b and CD45, and elevated production of inflammatory mediators following immune challenge [32].

A multitude of signals that pose a potential threat to brain homeostasis are sensed by microglial receptors [35,36]. Specific factors released by stressed or damaged neurons have the potential to stimulate the production of pro-inflammatory cytokines by microglia. These include matrix metalloproteinase-3 (MMP-3), α-synuclein, neuromelanin, and adenosine triphosphate (ATP). Danger signals emitted by necrotic cells that can stimulate similar responses include the heat shock proteins (HSP60, HSP70, HSP90, and gp96), the calcium-binding S100 proteins, DNA, proteases, uric acid, and the chromosomal protein high-mobility group B1 (HMGB1). Depending upon the stimulus, inflammatory responses can be initiated by pattern recognition receptors (PRRs) that include the Toll-like receptors (TLRs), the receptor for advanced glycation end products (RAGE), and scavenger receptors. In addition, microglia can detect ligands for CD40, CD91, and the intracellular NOD-like receptors (NLRs). These receptors initiate the signaling process by binding to pathogen associated molecular patterns (PAMPs). Ligation of PRRs leads to the activation of signal transduction pathways and regulation of diverse transcriptional and post-transcriptional molecules. These molecules include members of the nuclear factor kappa B (NF-κB), activator protein 1 (AP-1), and interferon regulator factor families, which modulate pro-inflammatory target genes encoding cytokines, chemokines, enzymes, and other molecules essential for pathogen elimination [37]. Microglial activation, in addition to being stimulus-dependent, is also likely to be a multi-step process which, at least for EAE, involves both CD40-independent and CD40-dependent stages of stimulation [38].

The TLRs are a major family of PRRs for a diverse set of novel pathogen-associated molecules [39]. These receptors bind highly conserved structural motifs, the PAMPs, which are essential for survival of the respective pathogen. TLRs and their related signaling proteins are expressed in the CNS [40,41], with microglia expressing TLR 1–9 and astrocytes *in vivo* expressing TLR3 [42]. Early *in vitro* work suggested that all glial cells expressed TLR2 [43], however, *in vivo*, TLR2 expression was exclusively in microglia activated with cerebral ischemia [44], or upon axonal injury due to an entorhinal cortex lesion [45]. Similar to other family members, TLR4, which recognizes a fragment of gram-negative bacteria called lipopolysaccharide (LPS), can initiate innate immune responses to infection in mammals. Microglia derived from human white matter and in primary rat cell cultures express TLR4 [46,47]. A large proportion of the data suggests that TLR signaling mediates beneficial effects essential for pathogen elimination. But there is additional data suggesting that TLR-induced activation of microglia and the release of pro-inflammatory molecules can contribute to neurotoxicity. Based upon studies examining the various TLRs, it was suggested that the activation of innate immune responses in the brain are tailored according to the cell type and environmental signal. As an example, TLR3 signaling induced a strong pro-inflammatory response in microglia as characterized by the secretion of IL-12, tumor necrosis factor-α (TNF-α), IL-6, CXCL-10 and IFNβ. TLR2-mediated responses were primarily associated with secretion of IL-6 and IL-1β [48]. More recently, PRRs have been found to be able to respond to endogenously-derived molecules, such as factors released from necrotic cells and by molecules that may be secondary to a pathogenic process. Additionally, they may serve to facilitate neuronal damage. In ischemic injury, there is evidence that TLRs, especially TLR2 and TLR4, are capable of sensing damage induced by ischemia and, as such, boost the pro-inflammatory response such that the infarct size is increased [44,49,50]. There are several host-derived ligands for TLR. One of these, HSP60, is released from dying CNS cells and, upon binding to microglia, it can induce TLR4 and MyD88-dependent secretion of potentially neurotoxic nitric oxide (NO) [51]. Similarly, necrotic neurons have been shown to activate microglia in a MyD88-dependent manner and that the subsequent pro-inflammatory response leads to an increased neurotoxic activity through the induction of glutaminase, an enzyme that produces glutamate [52]. This endogenous pathway may be common for various forms of neuronal injury and provide a linkage between CNS inflammation and neurodegeneration.

While TLR activation can contribute to neurotoxicity during CNS infection, there is evidence that TLR signaling can also mediate beneficial effects [53]. Microglia appear to be the major initial sensors of danger or stranger signals recognized by TLR4, and they secrete inflammatory mediators such as TNF-α and IL-1β. These cytokines can then act on astrocytes to induce a secondary inflammatory or growth factor repair response [54]. With an acute injury, the release of TNF from microglia may serve to counter any secondary injury [55], while the activation of microglia to remove cellular debris may serve to prevent subsequent tissue inflammation [56,57]. Microglia and astrocytes can detect Aβ through several sensors, including TLR4 [58], leading to the activation of signal-dependent transcription factors for downstream inflammatory response genes and clearance of the aberrant protein [59,60].

While TLR activation is a major inducer of neuroinflammation in the brain as a result of both infectious and sterile types of injury, research on interactions between ligand and receptor activation *in vivo* are in their infancy and caution is raised regarding the translation of data obtained from isolated cells in culture. The ability to distinguish unique cellular contributions or specific neurodestructive *versus* neuroprotective actions as a result of TLR activation is currently hindered by the lack of reliable antibodies and molecules capable of selectively inhibiting TLR signaling. Genetically modified mice deficient in the specific TLRs are useful for studying the general pathogenic role of the receptors in disease; however, more recent data demonstrating the potential redundancy of TLR pathways will limit the utility of the single TLR knockout mice in such studies.

Microglia and astrocytes express “scavenger receptors” that regulate the uptake of a number of substrates including oxidized proteins, lipids, and apoptotic cells, and may contribute to downstream cell signaling [61]. Expression of cytokine and chemokine receptors, potassium channels, various glutamate and gamma-aminobutyric acid (GABA) receptors, adrenaline and dopamine receptors, and purinergic receptors allows microglia to “sense” astrocyte and neuron activity, and coordinate tissue defense responses [62–64]. The expression of purinergic receptors, in particular, allows for a microglial response to ATP release upon cell death, traumatic injury, or ischemia [65]. Purinergic receptor activation helps regulate microglial release of pro-inflammatory cytokines, including IL-6 and TNF-α [66,67], and may act as a sensor for microglial phagocytosis [68].

RAGE, a cell surface receptor belonging to the immunoglobulin superfamily [69,70], is present on the surface of microglia, astrocytes, vascular endothelial cells, and neurons. Activation occurs with the production of advanced glycation end-products (AGEs) in pro-oxidant and inflammatory environments. RAGE contributes to the clearance of amyloid beta (Aβ) and is involved in apolipoprotein E (apoE)-mediated cellular processing and signaling [71]. RAGE recognizes other ligands, including serum amyloid A (SAA), S100 protein, and HMGB1. Increased production of these ligands is observed with cellular dysfunction and inflammation [72,73].

NLRs are soluble, cytoplasmic PRRs that act as sensors of cellular damage. In Alzheimer’s disease (AD), Aβ oligomers and fibrils induce lysosomal damage; this damage can then trigger NALP3 in microglia [74]. NALPs activate downstream signaling proteins, such as apoptosis-associated speck-like protein containing a caspase recruitment domain (ASC). This induces apoptosis, but it also contributes to the maturation of pro-inflammatory mediators like IL-1β and IL-18. In addition to peptide fragments, a decrease in cellular potassium concentration can activate NALP1 in neurons, leading to a similar activation of ASC, apoptosis, and IL-1β and IL-18 maturation. This process serves as the basis for the concept of the inflammasome, which may be centrally involved in regulating microglial neuroinflammatory responses to select pathogens.

Additional receptors that recognize apoptotic cellular material, such as phosphatidylserine on the inner membrane leaflet, are important for phagocyte clearance processes and may actually stimulate an anti-inflammatory response [75]. Recent identification of such receptors include T-cell immunoglobulin-and mucin-domain-containing molecule-1 (Tim4) [76], the metabotropic P2Y6 receptor that recognizes the nucleotide UDP released from injured neurons [68], and the triggering receptor expressed on myeloid cells-2 (TREM-2) [77]. *In vitro* studies examining TREM-2 signaling in microglia demonstrate a facilitation of debris clearance in the absence of inflammation [77]. A critical role for TREM-2 signaling has been demonstrated in polycystic lipomembranous osteo-dysplasia with sclerosing leukoencephalopathy, or Nasu-Hakola disease. This recessively-inherited disease is characterized by early onset dementia and may arise due to the inability of microglia to clear tissue debris *via* TREM-2 signaling [78]. The differential expression of these receptors in response to the intensity or stage of tissue injury, the type of injurious stimuli, or the presence of other soluble signals can exert significant control over the potency of the microglial response.

## 3. Microglial Pro-inflammatory Cytokines

From both *in vitro* and *in vivo* studies, activated microglia have been shown to produce numerous protein mediators. These include factors that are typically categorized as pro-inflammatory and anti-inflammatory cytokines. However, upon stimulation these cells also produce growth factors, chemokines, and neurotrophins, such as insulin-like growth factor 1 [79]. In addition, they can regulate the production of neurotrophic factors by neurons and other glia *via* cytokine production [80–83].

Cytokines are crucial mediators of the inflammatory response in the brain under pathological and chronic neurodegenerative conditions [84]. The primary cytokine molecules are IFNγ, TNF family members, lymphotoxin (LT)-α, and various interleukins (IL-1, IL-6, IL-8, IL-12 and IL-23). Many of the pro-inflammatory cytokines, such as TNF, IL-1, and IL-18, are synthesized as inactive precursor proteins that are processed by enzymatic cleavage into the final mature and biologically-active form. The active form of the protein is then capable of binding to its receptor to induce signal transduction processes. In addition to direct signaling for a pro-inflammatory event, the system employs numerous downregulatory events. These include induction of proteins that inhibit signal transduction pathways (e.g., SOCS proteins), induction of transcriptional repressors and transrepressors (e.g., ATF3 and Nurr1), as well as the production of soluble or cell-surface mediators with anti-inflammatory activities (e.g., IL-10, transforming growth factor (TGF)-β, resolvins, and ligands for TAM receptors). The diverse and multifunctional capacity of any given inflammatory molecule presents an additional challenge to understanding the cellular dynamics and impact of elevated expression. For example, IL-1α, IL-1β, and the IL-1R antagonist (IL-1RA) all work *via* activation (or antagonism) of IL-1 receptor 1 (IL-1R1), yet IL-1α and IL-1β can also elicit disparate IL-1R1-independent signaling events. IL-1β is a major regulator of the expression of several MMPs, it can induce the production of NO, and it can block glutamate uptake. However, it can also promote the production of growth factors and increase the deposition of extracellular matrix molecules, laminins, and chondroitin sulfate proteoglycans [81,85].

Using TNF-α as a pivotal, pro-inflammatory cytokine we can identify different features of the complicated response of CNS cells to multiple factors. Work from McGuire *et al.* [86] demonstrated that exposure of cultured embryonic rat mesencephalon neurons to TNF-α resulted in a dose-dependent decrease in the number of tyrosine hydroxylase (TH)-immunoreactive cells. Interestingly, the cell death was specific to the dopaminergic neurons and the study identified subpopulations of TH^+^ neurons that were resistant to TNF-α toxicity. The factors responsible for the selectivity of TNF-α induced toxicity for select DA neurons, but not other cell types in mesencephalic cultures, are not known. Differential sensitivity was suggested to reflect the expression of heterogeneous combinations of TNF-α receptor subtypes, which are themselves capable of signaling through diverse combinations of TNF-α-coupled signal transduction pathways present in different cell types. The dual and opposing effects of TNF-α within the CNS are well documented. For example, TNF-α can protect hippocampal and cortical neurons exposed to Aβ-peptide and, *in vivo*, can protect the CNS from excitotoxic, hypoxic, hypoglycemic, and traumatic insults [55,87–91]. On the other hand, a significant body of evidence also suggests that TNF-α can be neurotoxic [92–94].

## 4. Reactive Oxygen Species and Nitric Oxide

In parallel with the production of pro-inflammatory cytokines, microglial neuroinflammation is commonly associated with the production of reactive oxygen species (ROS) and NO-dependent reactive nitrogen species (RNS). ROS are oxygen-containing molecules that react with and oxidize vulnerable cellular constituents, including proteins, nucleic acids, and lipids. The brain is particularly vulnerable to the excess generation of ROS and RNS. In part, this is due to the large energy demands of neurons, the disproportionate consumption of molecular oxygen by the brain relative to the rest of the body, and an abundance of polyunsaturated fatty acids in neuronal membrane lipids, which are susceptible to free radical attack. In addition, the CNS has a relative paucity of antioxidant defenses and high levels of extracellular transition metals (e.g., iron and copper) capable of participating in free radical reactions. In neural tissues, oxidative stress can result in disrupted signaling processes and ion homeostasis, and is held accountable for events ranging from protein misfolding to the death of newly-generated neurons [95,96]. Superoxide, in particular, is implicated in microglial activation, cellular redox imbalance, and associated neurodegeneration. It is important to note that microglial production of superoxide exhibits significant species dependence as regards the quantity of superoxide produced in response to the same activating stimuli [97]. Thus, caution is emphasized in the translation of ROS production in experimental animals to responses in humans.

Endogenously produced ROS and RNS are an essential component of development and brain homeostatic processes in multiple cell types. During development, in addition to phagocytosis of apoptotic neurons, microglia-mediated respiratory burst may help to regulate the numbers of neurons integrated, in a nicotinamide adenine dinucleotide phosphate (NADPH) oxidase-dependent manner [98]. In mature tissues, ROS are commonly released by microglia to eliminate pathogens and elicit the controlled destruction of neuronal debris. Endogenous reactions facilitated by ROS and RNS in the healthy brain also include regulating neuronal excitation *via* redox-sensitive ion channels, increasing synaptic plasticity and associated memory function, influencing neurogenesis and neuronal differentiation, modulating immunologic responses, and controlling expression or activity of proteins involved in mediating cellular redox status, vascular tone, and the response to changes in extracellular oxygen concentration [99–102].

NADPH oxidase in endothelial cells, astrocytes, microglia, and neurons can contribute to the production of superoxide in the brain [102–104]. In general, superoxide production by myeloid cells is mediated by the phagocytic NADPH oxidase (NOX2), consisting of membrane-bound (gp91 and p22) and cytosolic (p47, p67, and p40) subunits, as well as a requirement for the GTPase, Rac, for full activity [105,106]. NOX2 is ubiquitously expressed in the brain [102], although higher levels may be found in microglia than in astrocytes or neurons. The microglial respiratory burst in response to certain tissue-disrupting stimuli involves Ca^2+^ and K^+^ channels and NADPH-dependent signaling to induce the release of superoxide [107–110]. Opening of cell surface ion channels on microglia, including P2X7, Kv1.3, TRPV1, and KCa3.1, causes an “acute phase” of activation, often involving protein kinase C (PKC)-dependent signaling to NADPH oxidase and downstream NF-κB prior to superoxide release and gene induction [102,111–116]. These types of channels have been shown to be specific for induction of ROS, as opposed to NO [115]. NOX2 can be activated in mononuclear cells by TNF-α, IFNγ, IL-1β, prion protein, ATP, and fibrillar Aβ, the latter of which was also shown to induce microglial proliferation and subsequent release of pro-inflammatory cytokines *in vitro* [116,117]. In addition, although phagocytosis can cause activation of NOX2, microglia do not uniformly undergo a respiratory burst when they initiate a phagocytic action [118].

The work of Barger *et al.* [119] suggested that glutathione (GSH) depletion and oxidative stress resulting from the NADPH-dependent respiratory burst induces glutamate release from microglia and that it is the elevation in this excitatory neurotoxicant that results in associated neuronal loss. Induction of NADPH oxidases has been shown to be involved in the neurotoxic response in various Parkinson’s disease (PD)-type models, including LPS, 1-methyl-4-phenyl-1,2,3,6-tetrahydropyridine (MPTP), rotenone, and paraquat, with a possible requirement for PKC delta phosphorylation of p67 and p47 [120–122]. Notably, activation of NOX2 alone is not always sufficient to cause neurotoxicity [123], suggesting that microglial superoxide production may have to occur in connection with other stressors to induce neurodegeneration. Some of these other factors that may contribute to neurodegeneration include reactive species (*i.e.*, hypochlorous acid and nitrites) produced *via* myeloperoxidase activity in astrocytes or peripheral leukocytes [103,124], and ROS-stimulated microglial proliferation and cytokine production [125,126]. *In vitro*, peroxynitrite formed from superoxide and NO demonstrates a greater level of toxicity than either radical alone [127,128]; it is unclear under what conditions and to what extent this short-lived molecule is produced *in vivo*, as methods for its direct measurement are virtually nonexistent [129].

In reaction to ROS, a cellular stress response is enacted in the brain. A key event in this response is the induction of heme oxygenase-1 (HO-1) in microglia and other cells, that can help protect against subsequent insults (e.g., [130,131]). This enzyme catalyzes the degradation of heme, resulting in the production of iron, biliverdin, and carbon monoxide (CO). Endogenously-produced CO helps to maintain the integrity of the cerebral vasculature and can enact protection against insults such as ischemia-reperfusion, excitotoxicity, and oxidative stress [132–135]. Although increased HO-1 activity and CO production can have neurotoxic effects in response to select insults, the regulated activation of this system in microglia is hypothesized to be involved in resolving neuroinflammation and preventing accessory tissue damage [136].

As opposed to ROS, which are almost exclusively, and in some cases inappropriately, viewed as detrimental to neurons, NO clearly occupies dual roles. NO freely diffuses across neuronal and vascular membranes and can initiate rapidly-induced and transiently-regulated signaling events. While NO-mediated stress is implicated in the progression of multiple brain insults, including AD, PD, and stroke [137], it can also induce neuroprotective signaling events. Either directly or indirectly, NO can cause inactivation of caspases, modulate release of neurotransmitters (e.g., dopamine, acetylcholine, GABA, and glutamate), and regulate synaptic plasticity by facilitating the induction of long term potentiation (LTP) *via* soluble guanylyl cyclase (sGC) [138,139]. In the brain, NO is produced from the conversion of the amino acid, L-arginine, to L-citrulline. It interacts with sGC, cyclooxygenase (COX) and HO-1, amongst other molecules, and can self-regulate, reducing the activity of nitric oxide synthase (NOS) [140]. Importantly, the functional role assumed by NO is strongly influenced by concentration, cell type, and NOS isoform.

Neuronal NOS (nNOS; NOS1) is found primarily in neurons, as well as astrocytes and endothelial cells of the blood vessels. Endothelial NOS (eNOS; NOS3) is the primary form found in endothelial cells, as well as some neurons, and contributes to neuroprotective vasodilatory properties attributed to NO. nNOS and eNOS are both Ca^2+^-dependent, constitutively active enzymes. In contrast, inducible NOS (iNOS; NOS2) is Ca^2+^-independent and can be upregulated in response to TNF-α and IFNγ, or downregulated in response to TGF-β and IL-1β [141]. iNOS is present in microglia, macrophages/monocytes [142], possibly neurons [143], and endothelial cells [139]. Activated astrocytes also express iNOS and can be stimulated to produce significant quantities of NO. However, there are notable differences in the cellular expression of iNOS across species [97,144]. Most notably, microglia in mice exhibit greater inducibility of iNOS, and subsequent production of NO, than those in humans. In addition to the species-dependent microglial production of ROS and NO, this process is also regulated in a stimulus-specific manner [97,145].

It is generally thought that, in response to stimuli, NO production is secondary to the oxidative burst, as it requires NF-κB-mediated gene transcription. Microglial NO can be cytoprotective and neuroprotective against select insults, such as ischemia [146,147]. It can also be cytotoxic, particularly at high levels, as shown with oligodendrocytes in culture and studies of direct effects on blood-brain barrier (BBB) permeability [148,149]. iNOS induction can also inhibit neuronal respiration, causing depolarization, glutamate release from neurons and astrocytes, and inhibition of cytochrome oxidase [117,150,151], eventually leading to excitotoxicity and/or synapse strengthening. While iNOS is often considered a major contributor to classical neuroinflammatory responses, as would be elicited by a pathogen, pro-inflammatory cytokine signaling in the absence of iNOS is proposed to represent an alternative activation process [152].

In various PD-like insults, iNOS is firmly implicated in the progression of neuropathology (e.g., [153]). In many cases, nNOS contributes significantly to toxicity (e.g., [122]), possibly through NO-mediated dopamine depletion [154]. In addition, anti-inflammatory agents that do not modulate NO production also show neuroprotection against MPTP [155]. In PD, ROS and NO overproduction can induce deleterious events including protein nitration/nitrosylation and misfolding, compromise of neuronal membrane integrity, and DNA damage, possibly as a secondary response to NMDA receptor stimulation, activation of NF-κB, and pro-inflammatory cytokine production [96,156]. The work of Farooqui *et al.* [156] suggested that excessive nitrosative stress underlies the initial hyperactivation of NMDA receptors, a potential key step in PD pathology.

## 5. Resident Microglia *versus* Infiltrating, Blood-Borne Monocyte Contributions

It is now generally acknowledged that all CNS disorders are characterized by microglial activation and that the progression and resolution of many diseases is contingent, in part, on the activity of microglia. In many of these clinical cases, such as stroke, head trauma, and advanced neurodegenerative disease, an associated disruption of the BBB is observed, allowing entry of cells from the bloodstream. In many of these conditions, it is possible that brain-resident microglia are not contributing significantly to many of the observed mononuclear cell effects. In addition to the secondary contribution of cells from the circulating blood, components of plasma such as fibrinogen can, in and of themselves, initiate a response in microglia [157–160].

The distinction between the source of the neuroinflammatory response, either resident microglia or infiltrating monocytes, becomes a critical issue in determining, not only the nature of the response and characteristics of the injury, but also the effectiveness of modulating their response. Cell trafficking *via* chemokine receptors and other adhesion molecules along vessels and natural barriers, such as the BBB, is required for the transport of myeloid cells. It is now recognized that microglia are derived from myeloid precursors and populate the CNS during development, with negligible turnover from postnatal hematopoietic progenitors or systemic mononuclear cells [5,161]. As there is no known cell-surface marker to distinguish brain-resident from blood-borne macrophages, it is difficult to discern the resident microglia from monocytes that enter the CNS from the bloodstream and subsequently adopt microglial-cell morphology [162]. Two approaches have been established to try to discriminate between resident and blood-borne macrophages and to track the engraftment of postnatal-derived hematopoietic microglia under physiological and pathological conditions. The first involves bone marrow chimera mice with labeled bone marrow cell replacement after irradiation to allow for tracking of infiltrating cells. The second approach relies on the observation that, in comparison with the macrophage population, parenchymal microglia express low levels of CD45 protein, a protein tyrosine phosphatase expressed by all nucleated cells of the hematopoietic lineage. In response to chronic pathology or acute, but robust, *in vivo* inflammatory signals, resident microglial CD45 levels increase, but only to levels intermediate between those of un-activated microglia and those of mature, circulating macrophages [163,164]. A combined flow-cytometry approach using the magnitude of CD45 in myeloid cells expressing CD11b, a pan-monocyte marker expressed similarly in both resident and non-resident populations, has been useful in determining the contribution of each cell type within an injured brain site. Both of these approaches, however, have some limitations, and thus, subtle contributions of highly-activated, circulating macrophages to changes within the brain parenchyma remain in question.

Initial studies using total body irradiation and transplantation of bone marrow suggested that the microglia pool within the brain received significant contribution from bone marrow-derived cells [165]. However, more recent studies employing additional controls for the effects of irradiation, or using new methodologies, showed no evidence of blood-borne microglia progenitor recruitment under either physiological conditions or in models of denervation or neurodegeneration [166,167]. These studies suggested that microgliosis is dependent upon local cell expansion and self-renewal, and varies as a function of the disease process. The recruitment of cells from the circulation occurs only under certain defined host conditions. In addition, engraftment of bone marrow-derived microglia in the absence of overt BBB disruption appears to require a level of prior conditioning of the brain [167].

In an effort to distinguish unique characteristics between resident microglia and infiltrating monocytes, Schmid and coworkers [168] conducted a detailed gene-expression analysis on cultured peritoneal macrophages or cultured microglia stimulated with IFNγ and LPS, and brain microglia isolated from an injection site of the same stimulus cocktail. In this study, the cultured microglia showed a gene profile more similar to the peritoneal macrophages when compared to the profile generated *in vivo*. Interestingly, if the resident microglia and infiltrating macrophages were separated by flow cytometry prior to gene profiling, the profiles from each cell population were similar. This observation led the authors to suggest that the CNS environment was the major contributor to the gene expression profile of mononuclear phagocytes. However, upon further work, the impact of each of these unique cell populations on neurons was identified to be significantly different, with the infiltrating peripheral macrophages inducing a significant level of cell death when co-cultured with hippocampal neurons [169]. The isolated, stimulated resident microglia did not induce neuronal death. Experiments examining the ability of mononuclear cells to recognize fibrillar Aβ peptides and clear amyloid plaques have demonstrated differential capacities of resident microglia and infiltrating blood-derived macrophages [170,171]. Other work has demonstrated that, with a traumatic brain injury, macrophages accumulate at the local wound site while microglia proliferate at sites peripheral to and distant from the wound site [172]. With ischemia, the elevation of TNF-α, IL-1β and NO within the ischemic core contributes to the secondary growth of the lesion, whereas cytokine induction at remote sites facilitates neuroprotection [173].

Transection of the facial nerve results in a rapid accumulation of microglia around the axotomized, ipsilateral brainstem nucleus of the facial nerve [174]. Early studies using bone marrow chimera mice suggested that the response was related to an infiltration of blood-borne cells; however, the lack of shielding the brain resulted in cranial irradiation and a permanent alteration of the cerebral vasculature [166,167,175]. This raised questions with regards to interpretation of the response in a non-irradiated animal. In a study examining localized hippocampal damage, a systemic injection of the neurotoxicant, trimethyltin, was used to initiate a brisk death of dentate granule neurons in the hippocampus. In this model, the microglial response was solely attributed to the resident microglia, based upon the lack of infiltration of fluorescent blood-borne monocytes in the bone marrow chimera mice [176]. In this case, the cranium was sufficiently shielded to prevent any localized brain irradiation. In addition, flow-cytometry for differential levels of CD11b and CD45 confirmed that the response within the hippocampus was associated with resident, and not circulating, macrophages (Kraft and Harry, unpublished observations). This model has allowed for the further examination of the resident microglial response and the heterogeneity of that response along a temporal and spatial progression, and has demonstrated that the production of TNF-α by resident microglia was critical for the pattern of toxicant-generated neuronal death [94].

## 6. Contribution of Microglia to Neurodegeneration

CNS neuroinflammation can have both detrimental and beneficial outcomes. One may consider that the rapid changes that occur with an acute trauma (e.g., the shift from near-absent to robust expression of several inflammatory molecules and immune subsets) could represent a detrimental process. Alternatively, it could be rationalized that the rapid upregulation of such factors represents a transient and coordinated host response that is necessary to mitigate the severity of the injury. The level of severity of a brain insult is tightly correlated with the robustness of microglial activation and the production of pro-inflammatory cytokines. Due to the observation that microglial activation is likely an early event in all forms of pathology, the presence of activated microglia was initially considered as a marker for future neuropathology [4]. However, further work has demonstrated that changes in microglia morphology or functional activation do not inevitably lead to neuron loss, nor does it only indicate damage.

It has been suggested that microglial responses are tailored in regional and insult-specific manners [169]. The most recognizable role of microglia in brain defense is as a scavenger of cellular debris by phagocytosis, as occurs in the event of infection, inflammation, trauma, ischemia, and neuronal death [177–180]. However, we now know that, not only do microglia dynamically survey the CNS and clear damaged cellular constituents, but that they are capable of initiating a rapid and specific response to subtle changes in the microenvironment. Different types of neuronal pathology and other activating stimuli clearly elicit differing responses from brain-resident microglia [152,181,182]. Some forms of brain injury may involve remodeling or destruction of specific regions of neuronal dendrites in response to changes in activity, neurite dysfunction, or excess extracellular neurotransmitter. In a similar manner, during development, the removal of excess excitatory synapses prevents the acquisition of epileptiform activity in mature animals [183]. Thus, in adulthood, this process of removing or “stripping” synapses and, in severe cases, remodeling the neurites themselves, is likely to be a protective mechanism in place to limit secondary neurodegeneration. Microglia monitor synaptic activity and contribute to remodeling of impaired synapses [184]. Recent studies have proposed a prominent role for microglia in mediating these types of actions in response to disrupted visual experience [185], in a mouse model of glaucoma [186], following mutant huntingtin-induced neurotoxicity [187], and as a protective response at mossy fiber synapses after trimethyltin exposure-induced DG neuron death (Kraft and Harry, unpublished observations). Although this theory requires further exploration [188], the available data supports that, in the vicinity of the neuronal nuclei that are presumed to require reorganization, microglia gain a reactive or bushy phenotype, display an altered expression profile of inflammatory cytokines, and may target neuronal constituents for destruction through recognition of complement proteins deposited on the aberrant synapses or neurites. Such rapid responses of murine microglia are diminished in models of neurodegeneration (e.g., [189]) and in the aged brain [190], where these cells show less motility and fewer processes, supporting the hypothesis of an impaired microglial functionality [191].

A contribution of microglial activation to neuronal death has been suggested in numerous reports of pharmacological downregulation of microglial activation by compounds such as minocycline; however, the potential neuroprotective effects are not uniformly observed across multiple injury models. This is possibly related to factors such as the source of the cells responding to the insult, given that minocycline can reduce BBB leakiness following Aβ injection [192], as well as leukocyte transmigration and microglial activation following traumatic brain injury [193]. Under conditions of intracerebral hemorrhage, the associated elevation of TNF-α within the brain is primarily due to neutrophils; a systemic injection of minocycline within 6 hours of the insult reduced TNF-α and MMP-12 expression, microvessel loss, and extravasation of plasma proteins and edema [194]. Effects such as these may occur independent of microglial activation.

Acute exposure to MPTP and dopaminergic neuronal loss is accompanied by microglial activation, however, activation is variable and difficult to detect when a subchronic exposure paradigm is used [195]. In acute exposure models of dopaminergic neuronal damage following MPTP and 6-hydroxydopamine (6-OHDA), protective effects of minocycline have been reported [196–198]. *In vivo*, a variety of studies have shown an associated increase in microglial numbers and reactivity following chronic administration of rotenone, paraquat, or maneb [199–203]. Interestingly, the timing of pre-exposure to LPS, and thus the status of microglia as primed or pre-conditioned at the time of paraquat exposure, significantly altered the neuronal outcome in that a seven day prior pre-treatment blocked neuronal cell death while a two day prior pre-treatment exacerbated the neuronal death [204]. Importantly, two compounds believed to selectively inhibit microglia, minocycline and iptakalim, have been shown to rescue nigral cell death following rotenone treatment [205]. However, in other models of more subtle brain disruption, such as administration of methamphetamine or trimethyltin [206,207], minocycline offers no level of neuroprotection and can actually result in a greater level of damage. While minocycline can downregulate microglial activation, it has alternative mechanisms of action related to direct anti-apoptotic effects and influencing BBB integrity [115,208,209], which may also significantly contribute to any neuroprotective actions. These series of studies raise questions with regards to the assumed mechanism of action of minocycline, as well as possible modulation of these actions dependent on the source of brain macrophages and the level of neuronal injury.

### 6.1. Chronic Neuroinflammation

Even in the absence of a prominent infiltration of leukocytes into the CNS, localized microglial activation can be observed in neurodegenerative diseases, such as PD and AD [210], and in manic depressive disorders [211]. The overall effect of microglial activation depends, in large part, upon the duration of the pro-inflammatory environment induced *via* extended production of cytokines, and the corresponding long-term receptor activation, or the absence of sufficient anti-inflammatory mediators to down-regulate the response. This persistent activation can produce a chronic inflammatory environment that can have detrimental effects on the surrounding tissue. Additionally, an underlying chronic inflammation may shift the impact of singular, acute inflammatory-related events. As an additional consideration is the possibility that dysfunctional or senescent microglia are less able to perform their normal beneficial roles and may fail to respond appropriately to immune stimuli [212]. Such a condition has been proposed as a function of aging, but a shift in the functional capability of microglia or astrocytes as immune-regulatory cells may underlie many disease states, not only in the adult, but also during brain development.

Much of the available data for chronic neuroinflammation has been derived from studies examining either human AD patients or in mouse models of AD, as well as data from prion disease. In the brains of AD patients, sites of amyloid plaques are cohabitated with clusters of activated microglia, suggestive of some inflammatory process. Murine models of AD have served to demonstrate that amyloid plaques form in the brain and become progressively larger, followed rather rapidly by the contact association of microglia [213]. Recently, the NALP3 inflammasome has been shown to mediate a fibrillar Aβ-induced microglial response. It is proposed that this occurs through a common innate immune mechanism that is shared across multiple insoluble aggregates [74], which is supported by the numerous reports of inflammatory proteins (e.g., acute-phase proteins, complement factors, and pro-inflammatory cytokines) that have been identified in AD brains [214]. Early work by Eikelenboom *et al.* [215,216] showed an absence of immunoglobulins and T-cell subsets in the neuropil, suggesting that humoral or classical cellular immune-mediated responses were not involved in plaque formation. The overall data suggests that, while fibrillar Aβ deposits are in close association with a locally-induced and non-immune-mediated, chronic inflammatory-type response, there is no evidence of an influx of leukocytes from the circulation. In comparison, the extracellular accumulation of amyloid fibrils of the prion protein (PrP) is associated with a neuroinflammatory response and microglial activation; however, in this case there is significant recruitment of blood-borne monocytes and T lymphocytes into areas of the scrapie-affected mouse brain [217,218]. Infiltrating cell contributions may account for the initial report of inflammatory microglia expressing IL-1, IL-6 and TNF-α in scrapie-affected brains [219].

The relative roles of Aβ and other potential initiators of inflammation remain unclear. However, one such role is the activation of caspases and signal-dependent transcription factors such as NF-κB and AP-1, resulting in the production of inflammatory factors such as IL-1β, TNF-α, IL-6. Under these conditions, these pro-inflammatory cytokines might directly act on neurons to induce apoptosis [220,221]. In addition, such pro-inflammatory cytokines released from microglia can act upon astrocytes and initiate the release of factors to further modify the surrounding environment or further activate microglia [54].

The contribution and role of microglia and neuroinflammation within the neurodegenerative disease process remains in question. One hypothesis put forth by McGeer and colleagues [222], the “inflammatory hypothesis” of AD, proposed that inflammation concurs with other neurotoxic mechanisms to cause the neuronal and synaptic pathology characteristic of AD. There is, however, an expanding body of literature suggesting that localized inflammation at a site of insult represents a protective mechanism. For example, the rapid clustering of microglia around Aβ plaques, and a co-localized increase in inflammatory markers, has been suggested to represent an effort aimed at reducing amyloid deposition through the normal cellular process of aberrant protein clearance. Experimentally, a positive role for neuroinflammation and microglial activation has been demonstrated in various transgenic mouse models showing that both complement activation and microglial phagocytosis are indispensable for amyloid clearance [223,224]. Using cultured microglia, Sawin *et al.* [225] demonstrated a dose-dependent effect of Aβ42 on the phagocytic actions of microglia that was regulated by lipid rafts. In addition, these investigators demonstrated that coexposure to the nonsteroidal anti-inflammatory compound, celecoxib, could inhibit phagocytic cup formation and, correspondingly, diminish the actions of microglia to phagocytize Aβ42. These data support a role for microglia in the clearance of excess Aβ and the potential for multi-directional regulation of microglial function with drug intervention. These experimental observations help in interpreting data available from clinical trials of anti-inflammatory compounds, including cycloxygenase 2 inhibitors, which have failed to show a distinct benefit in AD patients; rather, in a number of cases, the progression of the disease has worsened [226–228]. A contrasting view is that the immune system does not play a role and any changes observed are simply due to a bystander effect. In support of this, amyloid plaque formation and maintenance of amyloid-associated neuritic dystrophy was not altered in APP transgenic mice crossed with CD11b-HSVTK mice in which microglia were ablated *via* ganciclovir application [229]. Whether the actual plaques induce an inflammatory response is still in question [230]. However, it is generally accepted that, in AD brains, a discrete and localized inflammatory environment is induced nearby damaged neurons, neural fibrillary tangles, and amyloid deposits.

## 7. Examples of Application to Environmental Neurotoxicity

### 7.1. Manganese

The vulnerability of the nigrostriatal dopaminergic pathway and the role of this pathway in PD has been the focus of many studies examining the contribution of neuroinflammation to neuronal death. These efforts have focused on environmental agents that can produce similar clinical symptoms while sparing the nigral neurons (e.g., manganese; see [231]), or insults that lead to the loss of dopaminergic neurons (e.g., sepsis and rotenone) [232]. A role of neuroinflammation and associated microglial or astrocyte responses has been suggested for a number of these exposure models. However, in the case of rotenone, although microglial activation and elevated neuroinflammatory factors are often observed, the reproducibility and robustness of nigral degeneration is widely variable [233].

Excessive occupational exposure to Mn as a component of welding fumes and mining has been associated with neuronal damage in the globus pallidus, with less severe damage in the striatum and minimal damage in other basal ganglia structures, such as the substantia nigra, that are routinely affected in PD [234,235]. In addition, Mn-exposed patients do not respond well to the classic PD levadopa therapy [236–238], which may be related to both the lack of evidence of nigral neuron loss and damage to striatal or pallidal neurons possessing dopamine receptors capable of responding to the treatment. Clinically, Mn-induced parkinsonism is often associated with a high frequency, postural or kinetic tremor, but not the dyskinesia or resting tremor common in PD. Experimental models of primate exposure have confirmed a similar neuropathological profile with motor disturbances, neuronal loss and gliosis in the globus pallidus, and a lack of responsiveness to levodopa treatment [239]. Recent work using positron emission tomography (PET) in non-human primates reported a marked decrease in dopamine release associated with elevated brain Mn levels [240,241]. Further work suggested a selective effect of Mn on the substantia nigra pars reticulata (SNr), as compared to the substantia nigra pars compacta (SNc). Interestingly, in young (5–6 year old) non-human primates exposed to 5–6.7 mg Mn/kg body weight, 2 times/week/32–34 weeks, microglia within the SNc and SNr displayed retracted processes. In the SNr, microglia displayed morphology suggestive of a disintegration of distal processes [242]. The morphological pattern of dysmorphic microglia was similar to that observed in the aging brain and with neurodegenerative disorders [2,243], suggesting that Mn exposure may have an adverse effect upon microglia and thus significantly influence the microenvironment of the dopaminergic neurons.

Experimental rodent studies have attempted to model the human neurodegenerative effects of manganese; however, in many cases the rodent does not show sensitivity to manganese neurotoxicity and often fails to demonstrate the clinical signs relevant to the human and non-human primate. Recent work by Sriram *et al.* [244] suggested that direct pulmonary exposure of rats once a week for seven weeks to complex mixtures of welding fumes containing either high or low levels of manganese, resulted in pulmonary inflammation, cytotoxicity and, particularly with the high-Mn exposure, deposition of Mn within various brain regions, including the striatum and midbrain. Within one day post exposure, the midbrain showed a lower mRNA level for the dopamine D2 receptor and loss of TH protein with either exposure. While this TH loss appeared to be transient and recovered in the low-Mn group, this effect persisted in animals exposed to high-Mn fumes beyond 105 days post-exposure. In the midbrain, mRNA levels for the inflammatory factors, CXCL2, TNF-α, and IL-6 were unchanged in the high-Mn group and only TNF-α was increased in the low-Mn exposure group. In the striatum, however, mRNA levels for all three factors were elevated in both exposure groups. Interestingly, although the TH loss persisted, these inflammatory indicators were resolved by 105 days post-exposure. mRNA levels for NOS2 were elevated in both exposure groups in both the striatum and the midbrain, but NOS1, NOS3, COX-2, and HO-1 mRNA levels remained unchanged. A microglial response, as determined by increased mRNA levels for Emr1 (F4/80) and Itgam (OX42) in the midbrain, accompanied the TH loss in the striatum and midbrain of the high-Mn exposure group only. However, these indicators of microglial activation recovered by 105 days post-exposure, with the only change persisting alongside the midbrain TH loss being a decrease in GFAP levels for astrocytes. Of interest is the speculation that the changes observed, including cytotoxicity, were due to Mn exposure. As previous stated, there is no clear evidence in human subjects or non-human primates that moderate levels of Mn initiate degeneration of dopaminergic pathways [231]. Thus, this data may reflect species differences or a synergy of Mn with other components of the complex mixtures of welding fumes. In any case, these series of experiments demonstrate the complex nature of examining markers of neuroinflammation, gliosis, and neuronal alterations *in vivo*, reiterating the need for multiple markers, regions, and times of examination in any interpretation of such effects. Further studies examining the impact of inflammatory factors on manganese-induced dopaminergic neuron death have focused primarily on *in vitro* culture systems. Using a N9 microglial cell line Chang and Liu [245] reported that manganese could exacerbate LPS-induced NO production. Such data raises the possibility that manganese exposure can alter the homeostatic balance of the brain, resulting in a system that is primed or preconditioned to respond differently upon classical activation of the immune system. Additional work suggested that the increased production of pro-inflammatory cytokines by LPS-activated microglia exposed to Mn was associated with increased and persistent activation of p38 kinase [246]. Recent work using co-cultured astrocytes/microglia and neurons suggested that MnCl_2_ did not alter the number of TH-immunoreactive neurons until the concentrations reached 30 μM [247]. When co-cultures were exposed to both MnCl_2_ and LPS, a loss of TH^+^ cells was observed at a lower MnCl_2_ dose. Examination of the associated release of TNF-α, IL-1β, and nitrite revealed that MnCl_2_ exposure alone was not sufficient to elevate the protein levels, even at the 30 μM dose level for which TH^+^ cell loss was observed. LPS however, induced the production of TNF-α, IL-1β, and nitrite. The data at the high LPS dose level (2 ng/mL) suggested a synergistic effect of co-exposure to MnCl_2_, with a significant increase seen at MnCl_2_ levels of 3μM and above. Upon further examination, MnCl_2_ was found to significantly potentiate LPS-induced release of TNF-α and IL-1β in microglia, but not in astroglia. MnCl_2_ and LPS were also more effective in inducing the formation of ROS and NO in microglia than in astroglia. Additionally, MnCl_2_ and LPS-induced ROS and RNS generation, cytokine release, and dopamine neurotoxicity was significantly attenuated by pretreatment with the potential anti-inflammatory agents, minocycline and naloxone [247].

### 7.2. Diesel Exhaust Particles

Microglia-mediated neuroinflammation has been implicated in the pathology induced by exposure to particulate matter present in polluted air, of which diesel exhaust particles (DEPs) are a major component. Diesel exhaust contains greater than 40 toxic air pollutants, including known neuro-modulatory contaminants such as NO, CO, benzene, lead, and zinc. It should be noted that this pollution consists of, not only particulate matter (PM), but also significant amounts of other possible confounders such as ozone, LPS, tobacco smoke, and gasoline exhaust [248]. Work by Hartz *et al*. [249] suggested that exposure of isolated brain capillaries in culture to diesel exhaust particles (DEPs) produced an up-regulation of the efflux transporter, P-glycoprotein, *via* oxidative stress and TNF-α-dependent mechanisms. Direct exposure to DEPs (50 μg/mL), specifically, has been reported to decrease dopamine uptake in mesencephalic midbrain neuronal cultures 8–9 days post-treatment *in vitro*. This selective effect upon cultured dopaminergic neurons was reported to be dependent upon activation of microglia to elaborate superoxide *via* NOX in response to phagocytosis of DEP particles [250]. As would be expected with cells of the monocyte lineage, the phagocytic function of the cells was observed with the addition of DEPs to the culture media. A shift in the morphological phenotype of the microglia was observed within 6 hours of DEP exposure, consistent in timing with other literature examining the phagocytic uptake of fluorescent beads by cultured microglia. Phagocytes like microglia can be physically stimulated and will be activated to engulf any foreign material within the media. This can result in a cascade of microglial activation responses, including elaboration of pro-inflammatory cytokines. Even if maintained in the presence of other CNS cells and in the absence of known stimulatory factors, cultured microglia display a quasi-activated phenotype [163,251,252]. A similar pattern of susceptibility of dopaminergic neurons in culture dependent upon activation of microglia-like BV2 cells has been reported for nanosize titanium dioxide [253]. In the absence of a filtered DEP solution, the contribution of other contaminants in the media and the responses of other glia or neurons in these culture systems remains a concern. Since DEPs can absorb organic chemicals and metals from the surrounding environment, it is unknown how this property affects the integrity of *in vitro* systems, particularly as regards DA uptake and microglial activation. In addition, the treatment of cells with DEPs after shaking to remove microglia from the astrocyte monolayer produces a population of microglia in a significantly more activated state than would be found *in vivo*. Such a shift in the activation state is also observed with the use of the BV2 microglia cell line. Thus, further examination along these lines of investigation will bolster the ability to translate the effects observed *in vitro* to those that would occur *in vivo*. Identification of the underlying mechanisms will require a significant level of attention to detail and controls to determine specificity of the response. Support for similar effects occurring *in vivo* are provided by a limited number of studies. Campbell *et al.* [254] exposed mice (4 hours, 5 days/week for 2 weeks) to concentrated airborne particulates at a site near heavily trafficked highways in Los Angeles, CA. All animals were treated daily with intranasal instillation of ovalbumin to induce lung sensitization. Under these conditions, exposure to either ultrafine or combined ultrafine + fine particles increased NF-κB activation in isolated brain nuclear fractions. In the cytoplasmic fraction, IL-1α protein was increased under both exposure conditions, while TNF-α elevation was increased only with the combined particle exposure. However, caution should be applied when interpreting these results as indicative of particle-mediated activation of microglial inflammatory processes. For example, when treated with particulate matter collected from sites at varying proximity from traffic sources, immortalized macrophages displayed no traffic density-dependent elaboration of TNF-α or IL-6; rather, these responses appeared to depend more on the quantity and composition of endotoxin and transition metals contaminating these particles [255]. Gerlofs-Nijland *et al.* [256] exposed rats to 0.4 ppm ozone for 12 hours in a whole body inhalation chamber 24 hours prior to initiating nose-only exposure to diesel engine exhaust (DEE) for 6 hours/day; 5 days/week for 4 weeks. DEE exposure in the absence of pre-ozone exposure was not conducted. In these studies, TNF-α and IL-1α proteins levels were selectively elevated in the striatum. mRNA levels for TNF-α and TNFp55 receptor (TNFR1) were not altered by DEE exposure in any of the brain regions examined. In contrast with the earlier study by Campbell *et al.* [254], NF-κB activation was also not altered in any of the brain regions examined. In a study using Indian ink as a particulate matter (PM) donor, it was shown that a direct injection in the perivascular space led to scavenging of particles solely by MHCII^+^ perivascular cells, with no ingestion by pericytes, microglia, or other macrophages [257], suggesting a localized phagocytic response and the lack of penetration of the particles into the brain parenchyma or blood vessels. This appears to be consistent for DEPs, based on similar deposition across most, but not all, brain regions after exposure. Interestingly, in rats, CO (4,000 ppm) inhalation exposure for 15 minutes produced no evidence of neuronal pathology or astrogliosis within 1-hour post-exposure; however, evidence for reactive microglia was observed [258], suggesting a rapid response to exposure in the absence of cell death.

Air pollution may be associated with CNS inflammation and disrupted neural transmission [259,260]. Exposure to diesel exhaust, which makes up a significant portion of the air pollution present in a number of the Mexican cities from which animal and human cohorts have been examined, is associated with gliosis and brain damage in rats and humans [261,262]. In an early study, Calderon-Garciduenas *et al.* [263] examined cortical tissue of feral dogs of mixed breed, from less than one year to 12 years of age, with uncontrolled diet and genetic background, living in a highly polluted region (Southwest Metropolitan Mexico City, SWMMC), as compared with dogs from a less polluted region (Tlaxcala, Mexico). From these random cohorts, the authors interpreted the data of elevated NF-κB activation, iNOS levels, and astrogliosis to indicate an adverse effect of diesel particulate matter on the brain. In further examination of neuroinflammation in association with high air pollution, Calderón-Garcidueñas *et al.* [264] examined the brain following autopsy of human patients (between ages 2–45 years; average approx 25 years of age) who died suddenly. Subjects were from low exposure housing environments in Tlaxcala and Veracruz, Mexico (n = 12) or from high exposure housing environments in Mexico City, Mexico (n = 35). In this cohort, the high exposure group displayed evidence of BBB disruption, and increased GFAP, COX-2, IL-1β, and CD14 (an LPS receptor) levels in the olfactory bulb and in secondary sites including the frontal cortex and the substantia nigra. No changes were observed in the hippocampus. Histopathology showed a prominent increase in perivascular mononuclear cells and other indicators of vascular damage in multiple brain regions. Particulate matter did appear to penetrate the CNS and was observed within olfactory bulb neurons (in 4/35 subjects from the 2008 Calderón-Garcidueñas cohort), however, in other regions, its presence appeared to be restricted to the capillary and perivascular space, at least partly within or in contact with abundant mononuclear cells or mononuclear cell-ingested red blood cells (RBCs) [264,265]. Similarly, dogs in high pollution areas exhibit enlarged cortical, perivascular space and accompanying hypertrophy of surrounding astroglia, presumably activated to maintain barrier integrity [266]. Based upon magnetic resonance imaging (MRI), prefrontal white matter lesions, which were presumed to be neuroinflammatory in nature, were more frequent (56.5 *vs.* 7.6%) in MRIs of children from the cities representing high pollutant *versus* low pollutant areas (n = 23 and 13, respectively) [267]. Further examination of the brainstem from nine children from these localities indicated evidence of inflammation and pathology in the auditory nuclei and, in live subjects, a delay in brainstem auditory evoked potentials in relation to exposure [259].

Complicating the connection between exposure and effects on the nervous system are the known, non-CNS changes induced by diesel exhaust. These include cardiovascular and respiratory effects which appear to involve elevated systemic inflammatory responses, at least for responses to levels well above ambient concentrations [268]. Vascular function is influenced by air pollution, including vasoconstrictive effects that are enacted even in the absence of the particles themselves and may involve reduced NO [269–271]. Restricted blood flow to the brain can cause hypoxia and associated neurological events, including activation of resident microglia and elaboration of cytokines or ROS, which may be incorrectly attributed to responses associated with CNS-penetrating DEPs. Additionally, *in vitro* systems incorporating lung epithelial cells reveal that DEPs can alter lung barrier properties, including reductions in the tight junction protein occludin [272]; induction of MMP-1, NOX, and ROS [273]; and elaboration of IL-8, GM-CSF, and ICAM-1, the latter even after removal of the particles and independent of particle size [274]. At the BBB, these changes, particularly increased endothelial cell expression of occludins and ICAM-1, would indicate barrier dysfunction and could result in secondary activation of brain microglia. In studies such as this, pre-existing vascular pathology, as may exist due to atherosclerosis or coronary artery disease, should be considered as influencing susceptibility. This underlying pathology can influence the influx of infiltrating monocytes and lymphocytes, the activation state (increased hypertrophy and cellular density) of parenchymal cells, and sensitivity of specific neuronal populations, such as those in the substantial nigra [275–277].

Fine particulate matter, such as that isolated from diesel exhaust, has been reported to elicit production of 8-hydroxy-2'-deoxyguanosine and hydroxyl radical in isolated, *in vitro* systems and, in an immortalized microglia cell line, can reduce ATP and GSH, cause mitochondrial membrane depolarization and induce TNF-α and IL-6 mRNA expression, as well as alter genes associated with “oxidative stress” and innate immunity [278,279]. In this study, the PMs with the most robust effects (separated based on induction of NF-κB in respiratory epithelial cells) were identified as having higher concentrations of nickel and vanadium. It is possible that the transport of these metals to neurons or glia near to where PM deposits after exposure may disrupt function and/or induce activation. While interesting, the existing literature is insufficient to characterize the neuroinflammatory properties of brain resident cells as causative of neuronal pathology that may be related to diesel exhaust exposure; however, the data warrants consideration for future *in vivo* animal experiments and carefully designed epidemiological studies.

## 9. Conclusions

It is clear that glial cells participate in the process of neurotoxicity development in both chemical and environmental insults, with physical injury, and in neurodegenerative disease [280]. What is not known is exactly how to interpret the available data for a given situation in order to identify the mechanism as beneficial or detrimental. Identifying an elevation in pro-inflammatory cytokines or a structural morphological alteration in microglia is relatively easy; determining the overall effect of these changes and their underlying biological justification is a much more complicated effort, as is the identification of indirect and secondary consequences from cell-cell interactions. Of further concern is how to translate data obtained from cell culture systems, either cells in isolation or in co-culture with other glia and neurons, to what may happen within the *in vivo* environment. This is not only due to the somewhat non-physiological nature of isolated cells in culture, but also to the lack of dynamic interactions between the resident cells within the brain, communication between the brain parenchyma and the vascular system, and the various down-regulatory mechanisms that continually serve to maintain homeostatic balance.

Any single molecule can have a multitude of functions, with a competition between the beneficial and the detrimental features determining the final outcome. This outcome is also modulated by cell type, duration of expression, magnitude of the response, and the balance of other inflammatory molecules in both the extracellular and intracellular environment. The acquisition of a specific microglial phenotype in response to a given stimulus can vary depending on previously encountered signals and, at least in some cases, is reversible [281]. In addition, the stimulus initiating the response of microglia and the selective activation of particular, receptor-mediated signal transduction cascades over others will significantly impact the outcome. Whether microglia are simply one component of an injury response, or if there is indeed a causal relationship between microglial activation and neuronal death, synapse loss, and subsequent neurodegeneration, still remains in question. Correctly interpreting the role of neuroinflammation and observations of microglial reactivity/activation to assess the neurotoxicology of environmental agents will require, not only that these diverse actions and endpoints be examined under relevant exposure conditions, but also that the dynamics of ongoing processes occurring in other cell types of the brain, such as juxtaposed neurons and their synaptic endings, be considered.
